# A Murine Model of Waning Scrub Typhus Cross-Protection between Heterologous Strains of *Orientia tsutsugamushi*

**DOI:** 10.3390/pathogens11050512

**Published:** 2022-04-26

**Authors:** Nicole L. Mendell, Guang Xu, Thomas R. Shelite, Donald H. Bouyer, David H. Walker

**Affiliations:** 1 Department of Pathology, University of Texas Medical Branch, Galveston, TX 77555, USA; nlmendel@utmb.edu; 2 College of Osteopathic Medicine, Marian University, Indianapolis, IN 46222, USA; guangxu@marian.edu; 3 Division of Infectious Diseases, Department of Internal Medicine, University of Texas Medical Branch, Galveston, TX 77555, USA; trshelit@utmb.edu; 4 Department of Pathology, Center for Biodefense and Emerging Infectious Diseases, Center for Tropical Diseases, Sealy Institute for Vaccine Development, Institute of Human Infections and Immunity, University of Texas Medical Branch, Galveston, TX 77555, USA; dobouyer@utmb.edu

**Keywords:** *Orientia*, scrub typhus, intradermal, hematogenous model, sublethal, immunity, cross-protection

## Abstract

*Orientia tsutsugamushi*, the etiologic agent of the life-threatening febrile disease scrub typhus, is an obligately intracellular small coccobacillary bacterium belonging to the family Rickettsiaceae and is transmitted by the parasitic larval stage of trombiculid mites. Progress towards a vaccine for protection against scrub typhus has been impeded by characteristics of the pathogen and the infection. There are numerous strains of *O. tsutsugamushi* in the Asia-Pacific region with geographical overlap. In human cases immunity has been described as poor against heterologous strains of the pathogen, as well as short-lived against the homologous strain, with a mean antibody reversion rate of less than one year. Animal models of cross-protection as well as of deterioration of this cross-protection are needed to enhance understanding of transient immunity to scrub typhus. To build upon current understanding of this ineffective protection we sought to utilize our recently developed models, sublethal intradermal infection followed by challenge via ordinarily lethal hematogenous dissemination. Mice that were initially infected sublethally with *O. tsutsugamushi* Gilliam strain and were challenged with an ordinarily lethal dose of heterologous Karp strain were protected from death by a robust immune response at one month after the primary infection as evidenced by an abundance of mononuclear cellular infiltrates in target organs such as lung, liver, and kidney; maintenance of body weight; and low bacterial loads in the organs. Waning protection from lethal Karp strain challenge indicated by weight loss mirroring that observed in naïve mice was observed as early as 9 months after primary Gilliam strain infection, and higher bacterial loads, severe disease, and eventual death in some mice was observed after challenge with Karp strain at 14 months post-initial heterologous infection.

## 1. Introduction

Naturally acquired immunity to scrub typhus in humans was reported to be suboptimal as early as 1917, and reinfection was suggested by 1946 [1,2]. Strain heterogeneity of the causative agent, *Orientia tsutsugamushi*, was addressed as early as 1939, when human studies utilizing the administration of live Pescadores strain afforded short-term protection against the virulent Niigata strain [3]. A better understanding that heterologous protection was short-lived emerged in 1950 when human volunteers who had previously had scrub typhus, which was unlikely to have been caused by Gilliam strain due to the geographic strain distribution, were inoculated intradermally with *O. tsutsugamushi* Gilliam strain. Eight volunteers who had scrub typhus within the previous two months did not develop disease, whereas 11 out of 16 volunteers in whom 11–25 months had elapsed since their prior scrub typhus diagnosis developed disease of similar severity to naïve controls [4]. Two volunteers who were challenged with the homologous strain after three years, one natural-route and one needle-challenge, did not develop scrub typhus illness [4,5]. Immunity of human volunteers generated by infection with *O. tsutsugamushi* Gilliam strain and subsequent treatment after one week with chloramphenicol was shown to be effective to prevent symptomatic infection for up to 14 months against homologous challenge, but 2 of the 13 volunteers had detectable rickettsemia [6]. However, heterologous strain challenge caused mild illness with rickettsemia one month after primary infection, and when challenged after one year all volunteers developed scrub typhus disease [5].

Vaccines for scrub typhus which have been tested in humans and animal models do not stimulate heterologous strain protection, and provide only limited short-lived protection from death, but not protection from illness, upon homologous strain challenge [5,7,8,9]. Limited homologous strain protection from illness was observed during operation “Tyburn”, the attempt in the United Kingdom to propagate a scrub typhus vaccine for extensive field-trials during the Second World War. Accidental laboratory-acquired infection and subsequent illness were reported in laboratory personnel who had received full courses of the formalin inactivated vaccine, which was prepared from cotton rat lung infected with *O. tsutsugamushi* Karp strain [10,11,12]. This project emphasized the importance of antigenic conformation in protection and implied poor homologous protection by denatured antigens. However, the authors of that report asserted that the absence of lethality in the four vaccinated cases was an improvement from the three fatalities among five unimmunized laboratory infections observed during vaccine propagation. Field trials of the same scrub typhus Karp strain vaccine in present-day Myanmar did not decrease the incidence of scrub typhus [13]. These infections were likely caused by heterologous strains [14,15]. A formalin inactivated rat lung–spleen vaccine utilizing *O. tsutsugamushi* Volner strain (isolated in the Philippines) proved ineffective against heterologous strains during a human field trial in Japan as evidenced by the isolation of 17 strains of *O. tsutsugamushi* from scrub typhus cases (9 Volner vaccine/11 murine typhus vaccine recipients) during the course of the study [16]. 

Vaccines that have been tested in animal models for scrub typhus utilizing live, fixed, or replication-deficient *O. tsutsugamushi* have resulted in variable, time-dependent protection against homologous challenge and inadequate, waning protection against heterologous strains. Complete homologous protection of Balb/c mice immunized with gamma-irradiated *O. tsutsugamushi* was accomplished, whereas incomplete heterologous protection lasted only six months. Trivalent vaccination containing multiple strains of irradiated *O. tsutsugamushi* (Karp–Gilliam–Kato) elicited longer-term protection that lasted 6 months against Kato and Buie strain challenges and 12 months against Karp and Gilliam strain challenges [17,18,19]. Gerbils whose initial *O. tsutsugamushi* infection was treated with para-aminobenzoic acid were protected from both homologous and heterologous challenge 6-9 months later [20]. In a guinea pig model, cross-protection was observed after recovery from primary challenge with a heterologous strain of *O. tsutsugamushi*; however, duration of this immunity was not addressed [21].

Currently, the mechanism of short-lived heterologous immunity is poorly understood. The objective of this study was to establish and characterize a murine model of solid immunity to ordinarily lethal heterologous challenge and subsequent time-dependent waning protection for future use in mechanistic studies.

## 2. Results

To establish a model of heterologous cross-protection between different strains of *Orientia tsutsugamushi*, mice were initially infected with a sublethal dose of *O. tsutsugamushi* Gilliam strain. Later, mice were challenged with an ordinarily lethal dose (5 lethal doses 50% (LD_50_)) of *O. tsutsugamushi* Karp strain one, three, nine, 14, and 19 months following the initial Gilliam strain infection and were observed for signs of illness, and samples collected to assess bacterial loads and physiologic responses to infection to evaluate the duration of this protective immunity.

### 2.1. Loss of Protection and Development of Illness

**Figure 1:**
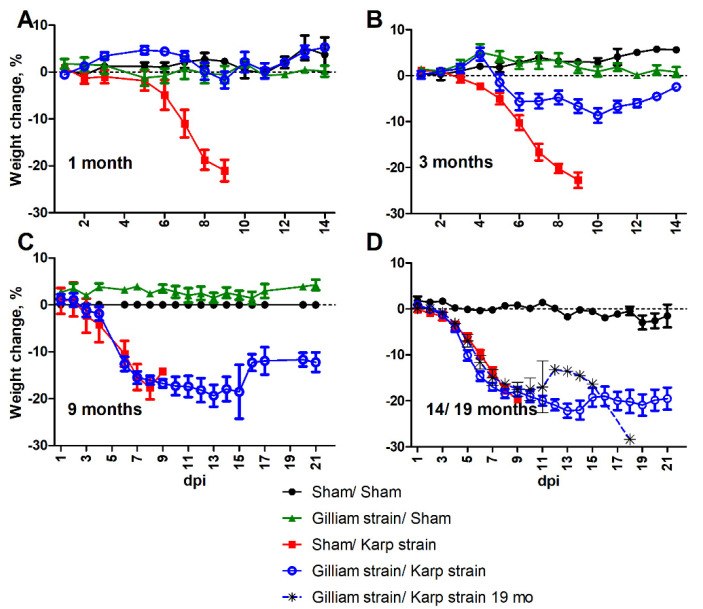
Weight change following challenge with a heterologous strain of *Orientia tsutsugamushi*. Percent body weight change of animals inoculated intravenously with 5 lethal doses 50% (LD_50_) of *O. tsutsugamushi* Karp strain at 1 (**A**), 3 (**B**), 9 (**C**), or 14/19 (**D**) month(s) after initial *O. tsutsugamushi* Gilliam (open blue circles) or sham inoculation (red squares 1, 3, 9, and 14 month(s)/black asterisks 19 months) as compared to sham/sham inoculated (closed black circles) or *O. tsutsugamushi* Gilliam strain/sham inoculated controls (green triangles).

Mice were protected from illness with no overt signs of illness or decrease in activity when challenged with an ordinarily lethal dose of *O. tsutsugamushi* Karp strain one month after infection with *O. tsutsugamushi* Gilliam strain. Body weight was maintained throughout the course of infection (Figure 1A). Three months after primary Gilliam strain infection, mice challenged with an ordinarily lethal dose of *O. tsutsugamushi* Karp strain exhibited labored breathing and slightly ruffled fur from six to nine days post-infection (dpi) as well as progressive weight loss from five to 10 dpi (Figure 1B).

**Figure 2:**
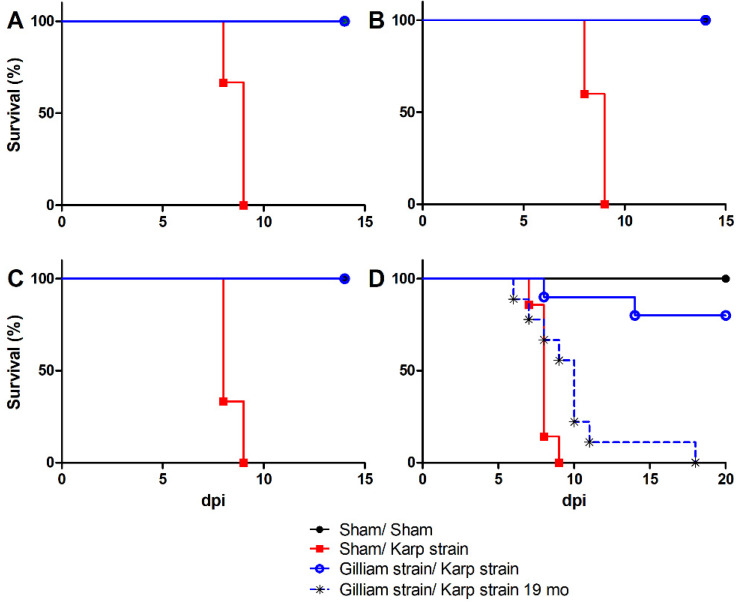
Time-dependent susceptibility to challenge with a heterologous strain of *O. tsutsugamushi*. Percent survival following sham inoculation (black circle) or inoculation with 5 LD_50_ *O. tsutsugamushi* Karp strain 1 (**A**), 3 (**B**), 9 (**C**), 14 (**D**), or 19 (black asterisk, **D**) month(s) after primary sham (red square) or primary infection with *O. tsutsugamushi* Gilliam strain (blue circle).

When mice were heterologously challenged with an ordinarily lethal dose of Karp strain nine months after primary Gilliam strain infection, they developed significant body weight loss concomitant with overt signs of illness including decreased activity, labored breathing, ruffled fur, hunched posture, and erythema beginning at six dpi and, conjunctivitis beginning at seven dpi (Figure 1C). The mean percent weight change observed at six dpi was −12.58% (SD = 3.99) and the mean nadir of −19.35% (SD = 4.64) on 13 dpi. Similar signs of illness were observed during this period in naïve lethally infected mice with a greater degree of decreased activity. The mean percent weight change for naïve lethally infected mice on six dpi was −10.42% (SD = 4.77) with the mean nadir of −17.60% (SD = 4.42) observed eight dpi. While all naïve mice succumbed to illness by nine dpi, heterologously challenged mice sustained erythema through 12 dpi, and ruffled fur and hunched posture through 14 dpi, and returned to normal activity by 16 dpi coincident with onset of weight gain. All heterologously challenged mice survived infection with Karp strain at one, three and nine months post primary infection with Gilliam strain, whereas all Gilliam strain naïve mice succumbed to infection between eight to nine days post Karp strain infection (Figure 2A–C).

Heterologous challenge 14 months post-Gilliam infection resulted in ruffled fur (10/10) and conjunctivitis (4/10) in Gilliam strain immunized mice beginning at four dpi, earlier than onset of overt illness in naïve mice, which was observed at five dpi. On five dpi signs of illness included erythema and hunched posture in all mice inoculated with *O. tsutsugamushi* Karp strain, but also labored breathing in heterologous challenged mice as well. At this timepoint, weight loss onset was observed by day three post-infection and the rate of decline matched for both groups infected with *O. tsutsugamushi* Karp strain and heterologously challenged mice that survived challenge had not recovered by day 21 post-infection (Figure 1D). Two (20%) of the heterologously challenged mice succumbed after inoculation with *O. tsutsugamushi* Karp strain 14 months after Gilliam strain infection (Figure 2B).

All of the mice succumbed (n = 9) to heterologous Karp strain challenge 19 months after the initial Gilliam strain infection. Decreased activity accompanied by ruffled fur was observed by day four following ordinarily lethal Karp strain challenge, and these observations expanded to include labored breathing, hunched back, erythema, and conjunctivitis by day five. The rate of weight loss for this group of mice typified lethal challenge of naïve mice (Figure 1D). All heterologously challenged mice succumbed to illness between days six and 18 following challenge (Figure 2B). 

### 2.2. Bacterial Dissemination in Previously Challenged Mice

**Figure 3:**
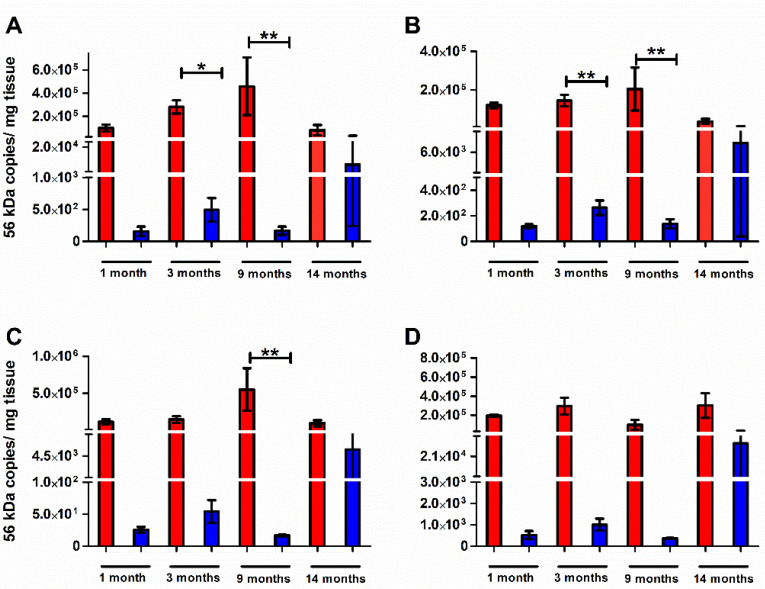
*Orientia tsutsugamushi* Karp bacterial burden following challenge. *O. tsutsugamushi* Karp specific bacterial loads expressed as genome copies (56 kDa gene) per milligram (mg) of tissue of spleen (**A**), kidney (**B**), liver (**C**), and lung (**D**) after *O. tsutsugamushi* Karp strain infection in naïve mice (red n = 6, day 7–9) or mice at 1 (n = 4), 3 (n = 6), 9 (n = 3), or 14 months (n = 2, day 8, 14) after *O. tsutsugamushi* Gilliam strain infection (blue). *, *p* < 0.05; **, *p* < 0.01.

**Figure 4:**
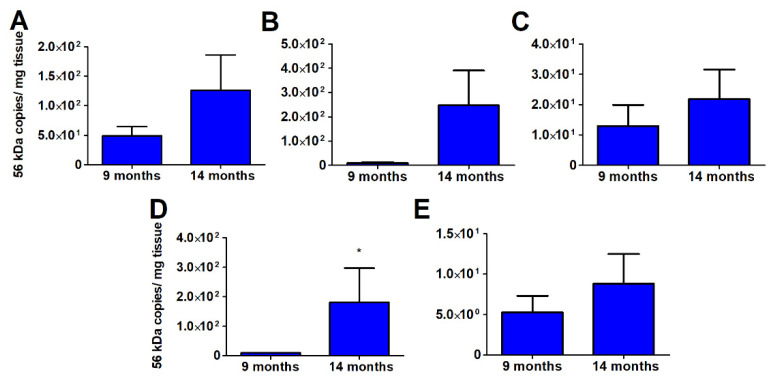
*Orientia tsutsugamushi* Karp bacterial burden following ordinarily lethal challenge. *O. tsutsugamushi* Karp specific bacterial loads, expressed as genome copies (56 kDa gene) per milligram (mg) of tissue, of spleen (**A**), kidney (**B**), liver (C), lung (**D**), and brain (**E**) 21 days after *O. tsutsugamushi* Karp strain challenge of mice at 9 (n = 3) or 14 months (n = 8) after *O. tsutsugamushi* Gilliam strain infection (blue). *, *p* < 0.05.

Tissue-specific bacterial loads indicate disseminated infection to spleen, kidney, liver, and lung after infection with an ordinarily lethal dose of Karp strain (Figure 3). However, in the previously Gilliam strain-infected group, the mean bacterial loads in the protected mice were markedly lower compared to the infected naïve mice. At the one-, three-, and nine-month timepoints when animals were completely protected from death, tissue bacterial load was the lowest in liver (Figure 3B) and highest in the lung (Figure 3D). An average of 1308-fold lower mean spleen bacterial load was observed in mice previously infected with Gilliam strain one-, three-, or nine- months prior (1,3,9 months-protected) than in infected naïve age-matched mice whereas the mean splenic bacterial loads for mice that succumbed to infection 14 months post-Gilliam strain infection (14-months succumbed) was only reduced 4.83-fold (Figure 3A). The same trends of reduction in bacterial loads for prior Gilliam strain infected mice were observed in the kidney (1020-fold 1-,3-,9- months protected/5.51-fold 14-months succumbed, Figure 3B), liver (13551-fold 1-,3-,9- months protected/20.4-fold 14-months succumbed, Figure 3C), and lung (313-fold 1-,3-,9- months protected/14-fold 14-months succumbed, Figure 3D). Mice that survived heterologous *O. tsutsugamushi* Karp strain challenge after 14 months cleared the infection more slowly than mice that survived heterologous challenge after nine months as evidenced by significantly higher bacterial load (nine-month M = 9.72 56 kDa copies/mg tissue and 14-month M = 181.50, *p* = 0.012) 21 days after Karp strain challenge in the lung, a major target organ (Figure 4).

### 2.3. Physiologic Response to Heterologous Challenge

**Figure 5:**
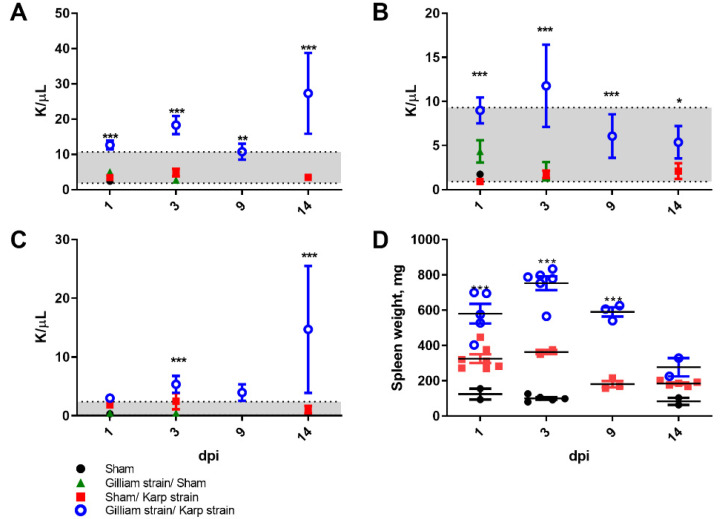
Hematologic responses to *O. tsutsugamushi* infection or heterologous strain challenge. Total white blood cell (**A**), lymphocyte (**B**), and neutrophil (**C**) counts expressed as thousand cells per microliter (K/µL) in whole blood or total spleen weight in milligrams (mg) (**D**) of mice 8–9 days post-infection with *O. tsutsugamushi* Karp (red squares) or heterologously challenged (open blue circles) with 5 LD_50_ *O. tsutsugamushi* Karp strain as compared to sham (closed black circles) or *O. tsutsugamushi* Gilliam followed by time-indicated sham inoculated controls (green triangles). Grey shaded region indicates normal range. *, *p* < 0.05; **, *p* < 0.01, *** or *p* < 0.001.

Hematologic analysis of mice between eight and nine days following heterologous challenge with an ordinarily lethal dose revealed a significant increase in white blood cells as compared to naïve Karp strain infected mice (Figure 5A) that comprised both elevated levels of lymphocytes (Figure 5B) and neutrophils (Figure 5C).

**Figure 6:**
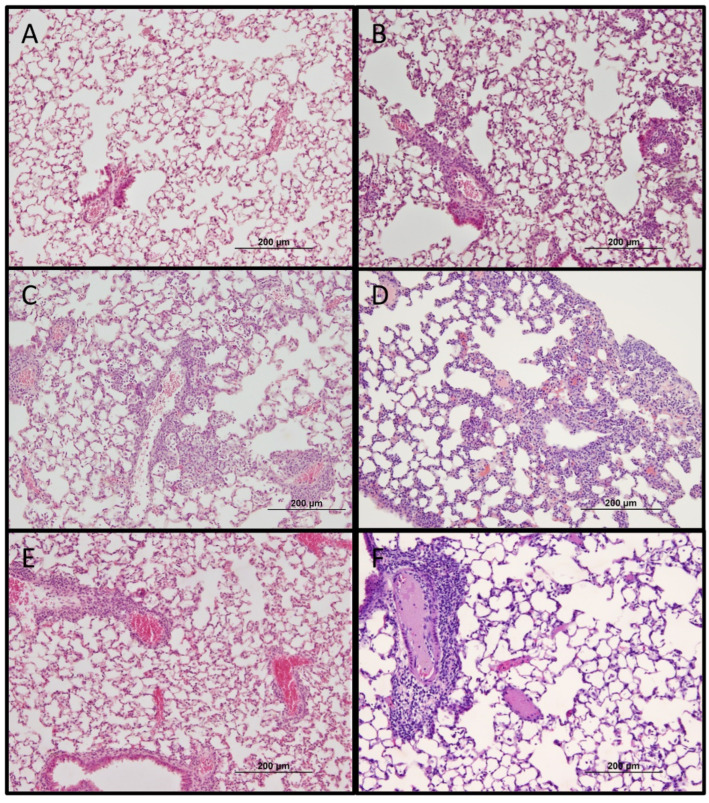
Histopathologic changes of lung one month following sublethal *O. tsutsugamushi* Gilliam strain infection (**A**) or day 8–9 after 5 LD_50_ inoculation with *O. tsutsugamushi* Karp strain in naïve mice (**B**) or 1 (**C**), 3 (**D**), 9 (**E**), or 14 (**F**) months after sublethal infection with Gilliam strain. Bar represents 200 µm (original magnification 100×).

**Figure 7:**
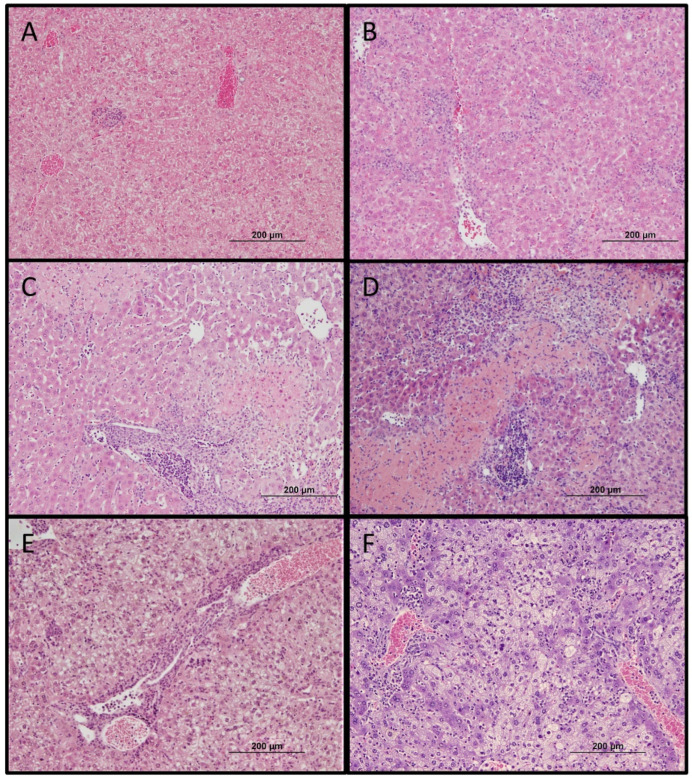
Histopathologic changes of liver one month following sublethal *O. tsutsugamushi* Gilliam strain infection (**A**) or day 8–9 after 5 LD_50_ inoculation with *O. tsutsugamushi* Karp strain in naïve mice (**B**) or 1 (**C**), 3 (**D**), 9 (**E**), or 14 (**F**) months after sublethal infection with Gilliam strain. Bar represents 200 µm (original magnification 100×).

**Figure 8:**
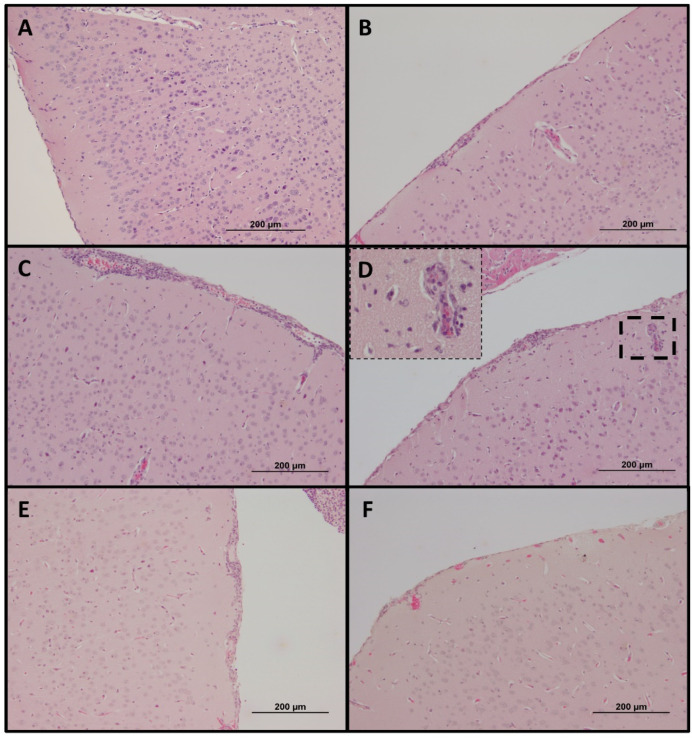
Histopathologic changes of brain one month following sublethal *O. tsutsugamushi* Gilliam strain infection (**A**) or day 8–9 after 5 LD_50_ inoculation with *O. tsutsugamushi* Karp strain in naïve mice (**B**) or 1 (**C**), 3 (**D**), 9 (**E**), or 14 (**F**) months after sublethal infection with Gilliam strain. Bar represents 200 µm (original magnification 100×).

Significant splenomegaly was observed in all mice that survived heterologous challenge as compared to naïve mice that succumbed to lethal *O. tsutsugamushi* Karp infection (Figure 5D). Histopathologic observations in lethally challenged naïve mice that succumbed to *O. tsutsugamushi* Karp strain included lymphohistiocytic vascular inflammation and interstitial pneumonitis (Figure 6B and Figure 7B). Although mice previously infected with Gilliam strain were protected from death, histopathologic changes, and lesions consistent with scrub typhus such as perivascular inflammation, multifocal lesions, and polymononuclear cellular infiltrates were observed in lungs, kidney, liver, and brain (Figure 6C–E, Figure 7C–E and Figure 8C,D). We observed coagulative necrosis in the liver of mice heterologously challenged three months after initial Gilliam strain infection (Figure 7D) and necrosis and steatosis in the liver of mice which succumbed to an ordinarily lethal Karp strain challenge 14 months after Gilliam strain infection (Figure 7F). Meningoencephalitis with perivascular inflammation was observed in the brains of heterologously challenged mice one and three months following primary Gilliam strain infection (Figure 8C,D). 

## 3. Discussion

We developed a murine model demonstrating waning protection against challenge with a heterologous strain of *Orientia tsutsugamushi* utilizing C57Bl/6 strain mice and a primary *O. tsutsugamushi* Gilliam strain infection followed by ordinarily lethal *O. tsutsugamushi* Karp strain challenge. In this model we observed no overt signs of illness after challenge with an ordinarily lethal dose of *O. tsutsugamushi* Karp strain one month after Gilliam strain infection; however, signs of illness were observed at three months following Gilliam strain infection and progressed in correlation with the period since the initial Gilliam strain infection. In addition to the loss of heterologous protection observed in this study, the use of age-matched mice at each challenge timepoint allowed us to observe that the kinetics and hematogenous dissemination of lethal infection with *O. tsutsugamushi* Karp was not affected by the increased mouse age.

Previous *O. tsutsugamushi* Gilliam strain infection does not result in sterile protection from Karp strain challenge in the animals that remained healthy as well as those that became ill. A fatal outcome was associated with greater bacterial loads. Mice that survived an ordinarily lethal challenge with *O. tsutsugamushi* Karp strain by having a previous heterologous infection had a robust immune response, characterized by reported pathologic manifestations of scrub typhus disease including splenomegaly and hepatomegaly. Although bacterial loads were relatively low in protected mice, an abundance of cellular infiltrates was observed in the tissues of protected mice which inversely correlated with the amount of time passed since the primary Gilliam strain infection. Immune-mediated damage and vascular inflammation have been described in a lethal murine scrub typhus model in the absence of high bacterial loads in tissues such as the brain [22]. Further investigation is warranted in this heterologous challenge model to understand the significance and mechanism of the histopathologic observations.

This model of waning protection between heterologous strains of *O. tsutsugamushi* will be used by our laboratory to study the immune components and cell subsets important for cross-protection. This model may also be employed to evaluate the difference between immunity to heterologous and homologous strains of *O. tsutsugamushi* Karp. In our laboratory, mice originally infected with a sublethal dose of *O. tsutsugamushi* Karp strain exhibited no overt signs of disease or significant weight loss upon re-challenge with a lethal dose (1.25 × 10^6^ organisms) of the homologous strain of *O. tsutsugamushi* 165, 180, 240, 292, or 430 days post-primary infection. In contrast, mice that survived Karp strain were heterologously challenged after 240 days with *O. tsutsugamushi* Gilliam strain which resulted in illness and weight loss. The protection observed from prior homologous or heterologous strain infection in these animal models indicate that native antigen is important to mount an effective immune response. For future vaccine development, consideration of current circulating human scrub typhus field isolates is paramount. This animal model provides the framework to develop future strategies for testing these isolates. We hypothesize that mouse strains that are less resistant to challenge with *O. tsutsugamushi* than C57Bl/6 may provide an opportunity to shorten the timeline of this model. Additionally, we will use the knowledge obtained from this model as a baseline to evaluate vaccine effectiveness and durability against heterologous strain challenge.

## 4. Materials and Methods

### 4.1. Stock Propagation

*Orientia tsutsugamushi* Gilliam and Karp strains were used in this study due to the availability of well-characterized sublethal and lethal murine models. The bacterial strains were obtained from the Rickettsial and Ehrlichial Species Collection at the University of Texas Medical Branch. *Orientia tsutsugamushi* was cultivated in L929 cells or harvested from mouse liver in vivo and stored at −80 °C in sucrose–phosphate–glutamate (SPG) buffer (218 mM sucrose, 3.8 mM KH2PO4, 7.1 mM K2HPO4, 4.9 mM monosodium L-glutamic acid, pH 7.0) until used as previously described [23,24]. The L-929 cell line was obtained from American Type Culture Collection (ATCC, catalog number CCL-1).

### 4.2. Bacterial Viability and Load Determination

A quantitative viability assay was utilized to enumerate viable *Orientia* for inoculation as previously described [25]. Bacterial loads and dissemination to selected organs were assessed by qPCR. Strain-specific primers were designed utilizing the variable domain IV of the 56 kDa gene (accession numbers DQ485289, AY956315, M33004) using PrimerSelect from Lasergene software suite version 12 (DNASTAR, Inc., Madison, WI, USA) (*O. tsutsugamushi* Gilliam OtG56.729 [5′-TCGTGATGTGGGGGTTGATAC-3′], OtG56.873 [5′-TTCTGAGGATCTGGGACCATATAG-3′], *O. tsutsugamushi* Karp 56 kDa OtK56.877 [5′-GATCCTAATGGGCCTATGGTTATA-3′], and [OtK56.982 5′-AACCTGCAGGCGGATTTG-3′]). DNA was extracted using a DNeasy Blood and Tissue Kit (Qiagen, Valencia, CA, USA) from bead-homogenized tissue samples according to the manufacturer’s instructions. Tissue samples were normalized using tissue wet weight, and bacterial loads were expressed as the number of *O. tsutsugamushi* Karp strain or Gilliam strain 56 kDa copies per milligram (mg) of tissue.

### 4.3. Mouse Infection

This animal study was approved by the Institutional Animal Care and Use Committee of the University of Texas Medical Branch-Galveston (protocol number 1302003 and approved 1 February 2013). Female C57BL/6 (B6) mice, six to eight weeks of age, were purchased from Envigo Laboratories (Indianapolis, IN, USA) and were housed in an animal biosafety level 3 facility (ABSL3) under specific pathogen-free conditions. The mice were allowed to acclimate for at least seven days prior to experimental use and then were inoculated intradermally in the lateral ear with 2.5 × 10^5^ *O. tsutsugamushi* Gilliam strain organisms as determined by viability assay and monitored twice daily for signs of illness for 2 weeks, and then weekly until secondary challenge. Mice were inoculated by intravenous tail vein at indicated timepoints post-Gilliam strain infection with 1.3 × 10^6^ viable *O. tsutsugamushi* Karp strain (approximately 5 LD_50_). Mice were monitored twice daily for signs of illness for up to three weeks. When mice were moribund, they were sacrificed humanely and necropsied along with matched mice from the other experimental groups, and their tissues were weighed, tested for bacterial loads, and prepared for histology. The remaining animals were observed for veterinary-accepted signs of illness (ruffled fur, hunched posture, erythema, lethargy, conjunctivitis, and weight loss). Moribund mice euthanized according to animal welfare criteria were counted as deceased for statistical analyses. All animal experiments were conducted twice.

### 4.4. Hematologic Analyses

Blood samples were collected at experimental endpoints in K_2_EDTA-coated BD microtainer tubes (Becton, Dickinson and Company, Franklin Lakes, NJ, USA) for hematologic analyses followed by centrifugation at 1300 RCF for 10 min. Blood cell counts were performed on whole blood using a calibrated 950FS HemaVet apparatus (Drew Scientific, Waterbury, CT, USA) using the FS-Pak reagent kit, and white blood cell count (WBC) and differential leukocyte (%) count were measured.

### 4.5. Histology

Tissue samples were fixed in 10% neutral buffered formalin (NBF) and embedded in paraffin. Tissue sections (5 µm thickness) were stained with hematoxylin and eosin and examined with an Olympus BX51 microscope (Olympus Scientific, Waltham, MA, USA).

### 4.6. Statistical Analysis

Values are reported as mean ± standard deviation (SD). The data were analyzed using a one-way ANOVA with Tukey’s multiple comparison as post hoc analysis or a two-way ANOVA with Bonferroni’s post-tests (GraphPad Prism, San Diego, CA, USA) and are reported at statistical significance levels of *, *p* < 0.05; **, *p* < 0.01; or ***, *p* < 0.001. 

## Data Availability

The data presented in this study are available in the article.
